# Supplementary material for: The Role of Inflammation in Anal Cancer
Source: Diseases. 2022 May 6;10(2):27. doi: 10.3390/diseases10020027 (PMC9149845; doi:10.3390/diseases10020027)
Supplement: Supplementary file 1 [file diseases-10-00027-s001.zip › diseases-1690794-supplementary.pdf]

Review

The Role of Inflammation in Anal Cancer

Amir Selimagic [1,*], Ada Dozic [2], Azra Husic-Selimovic [1], Nijaz Tucakovic [1], Amir Cehajic [1], Anela Subo [2], Azra Spahic [2] and Nedim Vanis [2]

1 Department of Gastroenterohepatology, General Hospital "Prim. dr. Abdulah Nakas", 71 000 Sarajevo, Bosnia and Herzegovina; husic_azra@yahoo.com (A.H.-S.); tucakovic.nijaz@gmail.com (N.T.); amircehajic@hotmail.com (A.C.)

2 Department of Internal Medicine, General Hospital "Prim. dr. Abdulah Nakas", 71 000 Sarajevo, Bosnia and Herzegovina; ada.dozic@gmail.com (A.D.); anelasubo@gmail.com (A.S.); azraspahicc@gmail.com (A.S.); nedim_08@yahoo.com (N.V.)

* Correspondence: selimagic.amir@gmail.com; Tel.: +387-33-285-100

Table S1. World Health Organization classification of tumors of the anal canal.

**Epithelial Tumors**
Premalignant lesions
Anal intra-epithelial neoplasia (dysplasia), low-grade
Anal intra-epithelial neoplasia (dysplasia), high-grade
Bowen disease
Perianal squamous intra-epithelial neoplasia
Paget's disease
Carcinoma
Squamous cell carcinoma
Verrucous carcinoma
Undifferentiated carcinoma
Adenocarcinoma
Mucinous adenocarcinoma
Neuroendocrine neoplasms
Neuroendocrine tumor (NET) (carcinoid)
NET G1
NET G2
Neuroendocrine carcinoma (NEC)
Large-cell NEC
Small-cell NEC
Mixed adenoneuroendocrine carcinoma (MANEC)
**Mesenchymal tumors**
**Secondary tumors**
